# Mesenchymal Stromal Cells for the Treatment of Graft Versus Host Disease

**DOI:** 10.3389/fimmu.2021.761616

**Published:** 2021-10-26

**Authors:** Kilian Kelly, John E. J. Rasko

**Affiliations:** ^1^ Cynata Therapeutics Limited, Cremorne, VIC, Australia; ^2^ Department of Cell and Molecular Therapies, Royal Prince Alfred Hospital, Sydney, NSW, Australia; ^3^ Gene and Stem Cell Therapy Program Centenary Institute, University of Sydney, Sydney, NSW, Australia; ^4^ Central Clinical School, Faculty of Medicine & Health, University of Sydney, Sydney, NSW, Australia

**Keywords:** graft versus host disease (GvHD), mesenchymal stromal (stem) cell (MSC), stem cell, bone marrow transplant (BMT), allogeneic

## Abstract

Graft versus host disease (GvHD) is a life-threating complication of allogeneic hematopoietic stem cell transplantation, which is initially treated with high dose corticosteroids. Approximately 50% of acute GvHD cases are resistant to steroid treatment, and two-year mortality rates in those steroid-resistant patients exceed 80%. Chronic GvHD necessitates prolonged corticosteroid use, which is typically associated with limited efficacy and troublesome adverse effects. No agent has yet been established as an optimal second line therapy for either acute or chronic GvHD, but mesenchymal stromal cells (MSCs) have shown substantial promise. MSCs promote an immunosuppressive and immunoregulatory environment *via* multifactorial mechanisms, including: secretion of proteins/peptides/hormones; transfer of mitochondria; and transfer of exosomes or microvesicles containing RNA and other molecules. A large number of clinical studies have investigated MSCs from various sources as a treatment for acute and/or chronic GvHD. MSCs are generally safe and well tolerated, and most clinical studies have generated encouraging efficacy results, but response rates have varied. Confounding factors include variability in MSC donor types, production methodology and dose regimens, as well as variations in study design. It is well-established that extensive culture expansion of primary donor-derived MSCs leads to marked changes in functionality, and that there is a high level of inter-donor variability in MSC properties. However, recent manufacturing innovations may be capable of overcoming these problems. Further adequately powered prospective studies are required to confirm efficacy and establish the place of MSC therapy in the treatment of this condition.

## Introduction

Allogeneic hematopoietic stem cell transplantation (HSCT) offers a potentially curative option for conditions including hematological malignancies. However, its benefits are often limited by serious complications, including graft versus host disease (GvHD).

GvHD arises from donor T‐lymphocytes attacking host tissues. Features of GvHD may be categorized as either acute or chronic, which were historically distinguished by the time of occurrence (<100 or >100 days post-transplant) (1). However, this is likely an over-simplification, because acute GvHD may persist beyond 100 days, and there may be overlap between acute and chronic syndromes (2). Both acute and chronic GvHD commonly affect the skin. Chronic GvHD is characterized by an initial lichenoid stage, similar to acute skin GvHD (3), often followed by a distinct sclerotic stage (4). Other organs typically affected by acute GvHD are the liver and gastrointestinal tract. Chronic GvHD may affect almost any organ in the body (5).

GvHD is the cause of death in 8‐16% of adult allogeneic HSCT recipients (6). It should be noted that these figures likely underestimate the extent to which GvHD contributes to post-transplant mortality, given that GvHD may also predispose HSCT recipients to other common causes of death, such as organ failure, infection and hemorrhage.

Acute GvHD is typically staged and graded according to criteria established at the 1994 Consensus Conference on Acute GvHD Grading (7). In most clinical trials, response to treatment is measured based on improvement in the severity of GvHD by at least one grade (Partial Response; PR) and/or resolution of all acute GvHD signs or symptoms, i.e. a return to Grade 0 (Complete Response; CR). The term Overall Response (OR) rate refers to the sum of PR and CR rates. Similarly, consensus criteria have also been developed for chronic GvHD, under which a global severity score is based on two different scores of the severity of cutaneous disease (8).

The prophylaxis and management of GvHD is complex, and approaches vary substantially between centers worldwide (9). Corticosteroids remain the mainstay of first-line treatment for both acute and chronic GvHD. Approximately 50% of acute GvHD cases prove to be resistant to high doses of steroids, and the prognosis in those patients is extremely poor, with two-year overall survival (OS) rates below 20% (10). In moderate-severe chronic GvHD, systemic steroid treatment for at least one year is typically required, with approximately 50-60% of patients requiring secondary “steroid-sparing” treatment (such as antithymocyte globulin (ATG), extracorporeal photopheresis (ECP) or mycophenolate mofetil), and more than 10% requiring systemic treatment for over seven years (11). Even when steroid treatment is effective in chronic GvHD patients, it may be associated with severe adverse effects, especially when administered systemically for lengthy periods.

Diverse second-line agents have been investigated for the treatment of GvHD after the failure of steroids. In 2019, the Janus kinase inhibitor ruxolitinib (Jakafi^®^, Incyte Corporation) was approved in the USA for the treatment of steroid resistant acute GvHD (SR-aGvHD) (12). In July 2021, the rho kinase (ROCK) inhibitor belumosudil (Rezurock™, Kadmon Pharmaceuticals) was approved in the USA, for the treatment of chronic GvHD after failure of at least two prior lines of systemic therapy (13). Both of these recent approvals apply to adults and children over 12 years of age only. Other agents investigated for acute GvHD include ATG, anti-CD26 antibodies, and ECP (14, 15). An even wider range of agents has been investigated for chronic GvHD, including Janus kinase inhibitors, tyrosine kinase inhibitors, proteasome inhibitors, monoclonal antibodies, and fusion proteins (16). However, to date no agent has been established as an optimal second line therapy for either acute or chronic GvHD, and there remains a need for new therapies with superior safety and efficacy profiles.

The subject of this review is the use of mesenchymal stromal cells (MSCs) in the context of GvHD. Over the past two decades, there has been extensive interest in the potential therapeutic use of MSCs in a wide range of clinical settings (17), including in support of HSCT (18) and in the treatment of GvHD (19).

## Mechanism of Action

**Figure 1:**
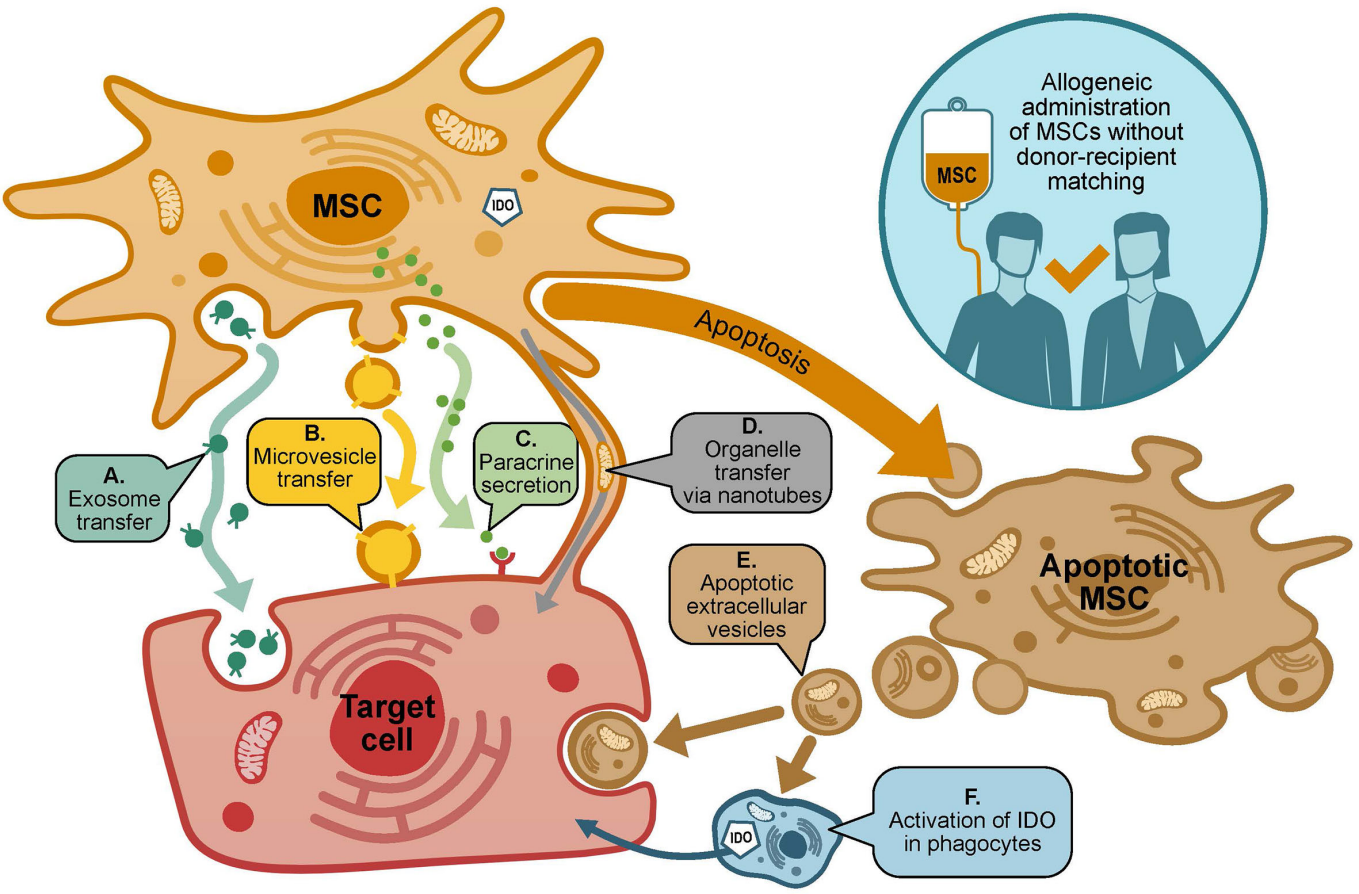
Mechanisms of action of MSCs in GvHD. MSCs may exert many effects on target cells *via* diverse potentially-overlapping mechanisms. Target cells include (i) donor and host immune cells, including T cells, B cells, NK cells, monocytes and dendritic cells; and (ii) host cells susceptible to damage by GvHD, e.g. cells of the skin, gastrointestinal tract and liver. Potential mechanisms through which MSCs may act include **(A, B)**: transfer of exosomes or microvesicles containing RNA and other molecules; **(C)** paracrine activity including secretion of proteins (including IDO), peptides and hormones; **(D)** transfer of organelles *via* tunneling nanotubes; **(E, F)** MSC apoptosis results in the release of apoptotic extracellular vesicles that act on target cells, as well as induction of IDO production in recipient phagocytes.

MSCs lack human leucocyte antigen (HLA) Class II expression, which allows allogeneic administration without donor-recipient matching. MSCs exert multifactorial effects, including: paracrine activity involving secretion of proteins/peptides and hormones; transfer of mitochondria by way of tunneling nanotubes or microvesicles; and transfer of exosomes or microvesicles containing RNA and other molecules (**Figure 1**) (20, 21).

MSCs promote an immunosuppressive and immunoregulatory environment, by secretion of cytokines, chemokines, growth factors and extracellular vesicles (22, 23). Notably, MSCs constitutively secrete indoleamine 2,3-dioxygenase (IDO), and activation of MSCs by inflammatory cytokines including interferon-gamma (IFN-γ) and tumor necrosis factor-alpha (TNF-α) leads to upregulation of this IDO secretion (24, 25). IDO, in turn, leads to suppression of allogeneic T cell proliferation (26). Further immunomodulatory effects of MSCs are mediated *via* effects on B cells, natural killer cells, monocytes and dendritic cells (24). An interesting idea is that apoptosis of MSCs *in vivo* contributes to their immunomodulatory effects, a phenomenon that may be mediated through the production and release of apoptotic extracellular vesicles (27). Additionally, when undergoing apoptosis, MSCs induce IDO production in recipient phagocytes (28). It has also been shown that amelioration of GvHD in a humanized mouse model was associated with altered phosphorylation and cellular localization of the T cell-specific kinase, Protein Kinase C theta (PKCθ) (29).

Aside from immunomodulation, MSCs have also been shown to limit tissue damage and stimulate tissue repair, primarily as a result of paracrine effects on other endogenous recipient cells (20).

## Clinical Experience

The use of MSCs to treat GvHD in a human subject was first reported in 2004 by Le Blanc *et al*, of Karolinska Institutet, Sweden (30). After receiving an allogeneic HSCT from a HLA-matched, unrelated donor, a nine-year-old boy developed Grade IV acute GvHD, which was refractory to treatment with corticosteroids and several second-line agents. The authors reported that the other 24 patients at their center who had experienced such severe acute GvHD had all died within 6 months, with a median survival of just 2 months. In this case, the patient was treated with two intravenous (IV) infusions of bone-marrow-derived allogeneic MSCs (on Days 73 and 150 post HSCT) from a related donor (his mother). The first infusion was followed by a marked but incomplete improvement, while after the second infusion the patient appeared to have recovered completely, and he remained alive and well after one year.

Since that initial case report, numerous clinical trials have been conducted to investigate MSCs as a treatment for GvHD. As of 30 June 2021, a total of 43 interventional clinical trials/expanded access programs involving MSCs as a treatment for GvHD have been registered on clinicaltrials.gov. Of those studies, 19 are complete, and papers summarizing the results of 10 have been published. Papers summarizing a further 17 studies involving patients with GvHD that were not registered on clinicaltrials.gov have also been published (studies conducted outside of the USA are not required to be registered on clinicaltrials.gov). **Table 1** summarizes the overall characteristics of the published and unpublished studies registered on clinicaltrials.gov.

**Table 1:** Overall Summary of Characteristics of Clinical Studies of MSCs in GvHD (n = 60).

Study Type	n	Age group	n	MSC Source	n
Phase 1	12	Adults only	23	Bone marrow	42
Phase 1/2	15	Adults and children	32	Cord blood	7
Phase 2	13	Children only	5	Adipose tissue	2
Phase 2/3	4			iPSCs	1
Phase 3	6	**Registered on clinicaltrials.gov**	43	Not stated	8
Compassionate use	10	Not yet recruiting	3		
		Recruiting	5	**MSC Donor Type**	
**GvHD Type**		Active, not recruiting	1	Allogeneic	1
Acute GvHD	40	Withdrawn/terminated	2	Autologous	57
Acute or chronic GvHD	10	Complete	19	Not stated	2
Chronic GvHD	10	Unknown	13		

The vast majority of studies have involved allogeneic MSCs, with bone marrow being the most common tissue source. A small number of trials have used MSCs derived from cord blood or adipose tissue, and a single study to date has been undertaken using iPSC-derived MSCs. The trials have been conducted by a wide range of sponsors, including both academic and commercial institutions.

As summarized in **Table 2**, most (n=18) published studies included only patients with SR-aGvHD. The patient population varied across other studies: five included patients with either SR‐aGvHD or chronic GvHD; two included only patients with chronic GvHD; one included patients with either SR-aGvHD or newly diagnosed acute GvHD; and one included only patients with newly diagnosed acute GvHD.

**Table 2:** Summary of Published Clinical Studies of MSCs in GvHD.

First-line Treatment of Acute GvHD, in Combination With Corticosteroids								
Reference	Study Type^a^	MSC source [dose^b^] (# of infusions]	Group (if applicable)	D28 Response		OS		
				CR		OR		
Kebriaei et al. (31)	Phase 2 CT (n=32; age: 34-67)	BM [2 or 8] (2)	Low dose	88%		88%		69% (D90) (pooled cohorts)
			High dose	67%		100%		
Soder et al. (32)	Phase 1 CT (n=5; age: 35-63)	UCB [2 or 10] (2)	Low dose:	50%		100%		67% (D180)
			High dose:	33%		33%		33% (D180)
**Treatment of SR-aGvHD**								
**Reference**	**Study Type^a^**	**MSC source [dose^b^] (# of infusions]**	**Group (if applicable)**	**D28 Response**		**Response (other timepoints)**		**OS**
				**CR**	**OR**	**CR**	**OR**
von Bonin et al. (33)	Compassionate use (n=13; age: 21-69)	BM [0.6-1.1] (1-5)		8%	54%			31%*
Lucchini et al. (34)	Compassionate use (n=8; age: 4-15)	BM [0.7-1.6] (1-2)		24%	71%			63% (1y)
Muroi et al. (35)	Phase 1/2 CT (n=14; age: 4-62)	BM [2] (8-12)		75%	93%			57% (2y)
Introna et al. (36)	Phase 1 CT (n=40; age: 1-65)	BM [1 ± 0.5] (≥2)		28%	68%			50% (1y); 38% (2y)
Zhao et al. (37)	Phase 2 CT (n=47; age: 14-54) BAT control	BM [1] (2-8)	MSCs:	36%	75%			45% (3y)
			Controls:	26%	42%			23% (3y)
Muroi et al. (38)	Phase 2/3 CT (n=25; age: 5-66)	BM [2] (8-12)		24%	60%			48% (1y)
Salmenniemi et al. (39)	Phase 1 CT (n=26; age: 2-66)	BM [2] (1-6)		27%	62%			42%*
Bader et al. (40)	Compassionate use (n=69; age: 1-78)	BM [1-2] (1-4)		32%	83%			71% (6 m)
Kebriaei et al. (41)	Phase 3 CT (n=260; age: 0-70) BAT control	BM [2] (8-12)	MSCs:	37%	58%			34% (D180)
			Controls:	32%	54%			42% (D180)
Kurtzberg et al. (42)	Compassionate use (n=241; age: 0-18)	BM [2] (8-12)		14%	65%			67% (D100)
Kurtzberg et al. (43)	Phase 3 CT (n=54; age: 0-17)	BM [2] (8-12)		30%	70%			69% (D180)
Bloor et al. (44)	Phase 1 CT (n=15; age: 21-66)	iPSC [1 or 2] (2)	Low dose:	13%	63%	50% (D100)	88% (D100)	88% (D100)
			High dose:	57%	86%	57% (D100)	86% (D100)	86% (D100)
Soder et al. (32)	Phase 1 CT (n=5; age: 48-73)	UCB [2 or 10] (2)	Low dose:	33%	100%			100% (D180)
			High dose:	50%	50%			50% (D180)
Prasad et al. (45)	Compassionate use (n=12; age: 0-15)	BM [2 or 8] (8-12)				17% (D32); 58% (D60)	67% (D32); 75% (D60)	58% (D100); 40% (2y)
Sánchez-Guijo et al. (46)	Phase 2 CT (n=25; age: 20-65)	BM [0.7-1.3] (2-4)				46% (D60)	71% (D60)	44% (1y)
Ringden et al. (47)	Phase 1 CT (n=8; age: 3-61)	BM [0.7-9] (1-2)				75%*	75%*	38% (2y)
Le Blanc et al. (48)	Phase 2 CT (n=55; age: 0-64)	BM [0.4-9] (1-5)				55%*	71%*	35% (2y)
Arima et al. (49)	Phase 1 CT (n=3; age: 39-64)	BM [0.5] (1)				0%*	33%*	0% (2y)
Perez-Simon et al. (50)	Phase 1/2 CT (n=10; age: 18-65)	BM [0.6-2.9] (1-4)				10%*	70%*	20%*
Herrmann et al. (51)	Phase 1 CT (n=12; age: 21-58)	BM [1.7-2.3] (2-19)				58%*	92%*	50% (3y)
Ball et al. (52)	Compassionate use (n=37, age: 0-18)	BM [0.9-3] (1-19)				65%*	86%*	51%*
Resnick et al. (53)	Compassionate use (n=50, age: 1-69)	BM [0.3-2.3] (1-4)				34%*	66%*	
von Dalowski et al. (54)	Compassionate use (n=58; age: 19-71)	BM [0.5-2.1] (1-6)				9%*	47%*	19% (1y); 17% (2y)
Dotoli et al. (55)	Compassionate use (n=46; age: 1-78)	BM [1-29.8] (1-7)				7%*	50%*	20% (1y); 17% (2y)
**Treatment of Chronic GvHD**								
**Reference**	**Study Type^a^**	**MSC source [dose^b^] (# of infusions]**	**Group (if applicable)**	**CR**		**OR**		**OS**
Ringden et al. (47)	Compassionate use (n=1; age: 27)	BM [0·6] (1)		No response		No response		0% (1y)
Lucchini et al. (34)	Compassionate use (n=5; age: 5-12)	BM [0.7-1.4] (1-4)		40% (D28)		80% (D28)		100%*
Perez-Simon et al. (50)	Phase 1/2 CT (n=10; age: 21-66)	BM [0.2-1.2] (1-4)		13%*		50%*		63%*
Herrmann et al. (51)	Phase 1 CT (n=12; age: 31-53)	BM [1.7-2.3] (2-19)		29%*		57%*		29% (1y)
Jurado et al. (56)	Phase 1/2 CT (n=14; age: 24-60)	UCB [1 or 3] (1)	Low dose:	57% (pooled cohorts) (1y)		67% (1y)		67% (1y)
			High dose:	80% (1y)		80% (1y)
Salmenniemi et al. (39)	Compassionate use (n=4; age: 37-63)	BM [2] (1-6)		No response		No response		25%* (3m)
Boberg et al. (57)	Phase 1 CT (n=11; age: 20-61)	BM [2] (6-9)		Not reported		55%*		82%*

a. No internal control group unless stated.

b. Dose expressed as 10^6^ cells/kg.

CR, complete response; OR, overall response; OS, overall survival; CT, clinical trial; BAT, best available therapy; BM, bone marrow; AT, adipose tissue; UCB, umbilical cord blood; iPSC, induced pluripotent stem cell; y, year; D, day.

* Timeframe for assessment not specified, and duration of follow-up varied between patients in some cases.

## Discussion

There is broad consensus that MSCs are generally safe and well tolerated (17, 21). None of the published studies of MSCs in GvHD reviewed here identified any significant safety issues. This is consistent with the wider experience of MSCs in the treatment of other conditions. A systematic review of MSCs in 55 clinical trials, in which 2,696 patients received MSC treatment, found an association between MSCs and transient fever, but not with acute infusional toxicity, infection, thrombotic/embolic events, death or malignancy (58).

Clinical studies of MSCs for SR-aGvHD in particular have generated encouraging efficacy results, but response rates have varied. For example, four studies of MSCs in SR-aGvHD have reported D28 OR rates exceeding 80% (32, 35, 40, 44) and a further three have reported D28 OR rates of at least 70% (34, 37, 43). However, a number of other published studies have reported lower D28 OR rates, ranging from 50‐68% (33, 36, 38, 39, 41, 42).

There has been even greater variability in D28 CR rates, with an overall range of 8-75% reported in SR-aGvHD patients (32–44). Notably, CR and OR rates do not necessarily correlate: one study that reported a very high D28 OR rate (93%) also reported a very high D28 CR rate (75%), while another study reported a D28 OR rate of 100%, but a CR rate of just 33% (32, 35). While the latter study had a very small sample size (n=5), this inconsistency was also evident in larger studies: in a compassionate use study with the commercial MSC product remestemcel-L (n=241) the D28 OR and CR rates were 65% and 14% (i.e. 21% of responders were complete responders) (42); while in a Phase 3 clinical trial with the same product (n=260), the D28 OR and CR rates were 58% and 37% (i.e. 64% of responders were complete responders).

Overall, outcomes with MSCs compare favorably to those reported with other second-line agents. In clinical trials in patients with SR-aGvHD, ruxolitinib treatment led to D28 OR rates of 55-62% and D28 CR rates of 27‐34% (59, 60); etanercept treatment led to D28 OR rates of 50-53% and D28 CR rates of 0‐20% (61, 62); while one-month OR and CR rates in patients treated with ECP were <50% and 33%, respectively (63). It should also be noted that some safety concerns have been associated with ruxolitinib and etanercept.

Caution must be exercised in comparing results between studies. Confounding factors include variability in MSC donor types, MSC dose per infusion, and number of infusions per patient – even within the same trial in some instances. A further issue is that there is no universally accepted definition of steroid-resistance. Clinical trials in SR-aGvHD typically require patients to have failed to respond despite treatment with corticosteroids, but the minimum period of treatment required varies between trials [e.g. 3 days (48) or 7 days (41)]. Additionally, many of the published studies have been compassionate use programs rather than formal, prospective clinical trials, while most of the clinical trials have been open-label studies with no control group.

Importantly, there has also been a lack of standardization on the timeframe for assessment of outcome measures. The most common timepoint to assess acute GvHD response has been 28 days, and for that reason D28 CR and OR rates are shown in separate columns in **Table 2**. However, some studies assessed CR and OR at different specified timepoints, while in many studies the timeframe for response assessment was not specified, meaning that a response at any time during follow-up was counted. In studies where response rates were assessed at more than one timepoint, there was a marked increase in response rates at later timepoints (44, 45). Consequently, D28 response rates cannot be compared with response rates at later timepoints, or response rates at unspecified timepoints.

Similarly, many studies have reported OS rates at the time of last follow-up, and the duration of follow-up per patient has typically varied both within and between studies. Furthermore, some studies have reported OS only at early timepoints such as D100 or D180, which are likely to be too soon to draw any conclusions.

Greater standardization in the design of future clinical trials would facilitate more robust evaluation of the efficacy of potential GvHD treatments. In recent years, D28 OR rate has been the primary endpoint in several Phase 2 and 3 clinical trials in SR-aGvHD, including those that supported FDA approval of ruxolitinib (43, 59, 60). Although not necessarily the primary endpoint, this outcome measure has also been reported in numerous other MSC trials, along with D28 CR rate (32–42, 44). Another important consideration is what, if any, control group to include. Until recently, as there were no treatments specifically approved for SR-aGvHD, the only ethical control options in SR-aGvHD trials were: a best available therapy (BAT) control group; an external control group; or no control group. Each of those options had limitations, but in light of the recent FDA approval, the possibility now exists to conduct trials in SR‐aGvHD with ruxolitinib as a control. A proposed Phase 3 trial of a monoclonal antibody-based treatment in SR-aGvHD was recently registered on clinicaltrials.gov, which aims to demonstrate that the investigational agent is superior to ruxolitinib based on D28 CR rate (64). A similar design may be suitable for late-stage trials of MSC products, while a non-inferiority design might also be sufficient to support approval, especially as MSCs appear to have a very good safety profile. However, the inclusion of a ruxolitinib control might remain challenging in multinational studies, as ruxolitinib is not yet approved for the treatment of SR‐aGvHD in the European Union or many other jurisdictions.

Another notable variable lies in the MSC manufacturing processes used. Academic studies have typically used minimally expanded bone marrow-derived MSCs, but most commercially-sponsored studies have utilized bone marrow-derived MSCs that were extensively expanded using industrial-scale processes.

The initial trial with a commercially produced bone marrow-derived product (remestemcel-L) in acute GvHD showed positive results (31). However, the results of that study are difficult to interpret in the context of other published studies, as it involved first-line treatment of acute GvHD in combination with corticosteroids, rather than treatment of SR-aGvHD. Additionally, the study did not include a control group, and first-line acute GvHD treatment with corticosteroids in the absence of MSCs has been shown to result in CR and OR rates as high as 69% and 78%, respectively (65). A subsequent randomized-controlled Phase 3 trial of remestemcel-L in patients with SR-aGvHD was completed in 2009, but with disappointing outcomes, which were belatedly published in 2020 (41). The trial found that remestemcel-L treatment led to significantly improved OR and durable CR rates in patients with liver GvHD. There was also a higher OR rate in children treated with remestemcel-L compared to controls. Nonetheless, the trial failed to meet its primary endpoint – there was no statistical difference between the durable complete response rate in patients treated with remestemcel-L in comparison to those treated with placebo. In more recent years, further trials with remestemcel-L in SR-aGvHD have been completed, including: two single arm, open-label clinical trials in adults and children in Japan (n=14 and n=25, respectively) (35, 38); a single arm, open-label clinical trial in children in the USA (n=51) (43) and a large compassionate use study in children in the USA (n=241) (42). Those studies have generated more positive results, but CR rates in particular have been mixed (24-75% in Japan, and 14-30% in the USA).

A number of suggestions have been offered to explain the apparent inconsistency in outcomes between trials. A review published in 2013 observed that the most striking difference between academic and commercial MSC treatments was the extent of MSC expansion – ranging from the production of 5-10 doses per bone marrow donation at academic centers, to 10,000 doses with remestemcel-L (66). There is a substantial body of evidence in the literature demonstrating that extensive culture expansion of bone marrow-derived MSCs leads to marked changes in functionality (67, 68). There is also evidence that clinical efficacy of MSCs is impaired even by modest levels of expansion (69).

It is well-established that there is a high level of inter-donor variability in MSC properties. For example, MSC gene expression, differentiation, proliferation and colony-forming capacity vary markedly between donors (67, 70). The susceptibility of MSCs to activation by IFN-γ and TNF-α, and the consequent upregulation of IDO expression and suppression of T cell proliferation, is also donor-dependent (24, 71, 72). With respect to processes that rely on isolation of MSCs from random donors, this variability may lead to an unpredictable variability in efficacy – between, and potentially within, studies.

It has also been suggested that cryopreserving MSCs and then administering the cells immediately post-thaw may impair their functionality (66). However, the same approach has been used in the majority of clinical trials involving allogeneic MSCs, many of which have generated positive results.

A number of groups have attempted to circumvent the challenges associated with inter-donor variability and extensive MSC expansion using novel manufacturing approaches. One such approach is to generate an MSC bank from pooled bone marrow donations from multiple donors. There is evidence that this approach, known as the “MSC‐FFM” method, can facilitate consistency within an MSC bank, with encouraging clinical trial results (40). By pooling donations, a larger quantity of MSCs can be produced compared to a single-donor bank with a similar level of expansion. However, based on the upper end of the dose regimen range used in the initial clinical trial, we calculate that each bank would only suffice for the treatment of approximately 175 patients. There is also a need to investigate consistency between banks produced using this method.

An alternative approach is to rely on pluripotent stem cells (PSCs) as a starting material for the production of MSCs. PSCs have the capacity to replicate indefinitely without loss of pluripotency, in addition to the ability to differentiate into any adult cell type. This means that a single bank of PSCs has the potential to give rise to an effectively limitless number of therapeutic cells. There are two types of PSCs: embryonic stem cells (ESCs) and induced pluripotent stem cells (iPSCs). While both types of PSCs have broadly similar properties, research and commercialisation of ESC-based therapies has been hampered by ethical controversy and political/funding constraints. These issues do not apply to iPSCs, which are derived from adult cells. The generation of human iPSCs was first reported by two independent groups in 2007 (73, 74). To illustrate their enormous self-replication capacity, it has been reported that even after 10^(71)^-fold expansion in culture, iPSCs retain their ability to differentiate into all three germ-layers (75). We have conducted a Phase 1 clinical trial in SR-aGvHD with iPSC-derived MSCs produced using a proprietary process (Cymerus™, Cynata Therapeutics Limited) (44). In contrast to processes reliant on the isolation of primary MSCs from donated tissue, a single iPSC bank has the capacity to produce 29 million clinical doses (each containing 1x10^8^ MSCs) using this process, at current scale. Thus, problems associated with inter-donor variability would be virtually eliminated. Furthermore, as this process achieves its scale by expansion at the iPSC stage, and prior to differentiation of the cells into MSCs, it involves relatively little expansion at the MSC stage. This is expected to minimise the type of functional changes that have been observed after extensive expansion of primary MSCs.

The small number of published studies in chronic GvHD comprise compassionate use studies or Phase 1/2 clinical trials, and all have had small sample sizes (n=1-14). Results have been mixed, with responses of 0-57% (CR) and 0-80% (OR), at various, and in some cases unspecified, timepoints (34, 39, 47, 50, 51, 56, 57). It is difficult to draw conclusions from this limited dataset.

As represented in **Figure 1**, multifactorial effects of MSCs have been identified, which include transfer of exosomes, microvesicles and organelles, and paracrine activity mediated by secretion of immunomodulatory molecules (20–26). In recent years, it has been suggested that the immunomodulatory effects of MSCs result in part from apoptosis, and the subsequent release of apoptotic extracellular vesicles and activation of IDO production in macrophages (27). In addition to the fact that MSCs act in numerous different ways, a further complication is that MSCs target a wide range of cells *in vivo*. In the context of GvHD, the target cells fall into two main categories: (i) immune cells from the host and HSCT donor; and (ii) cells that are damaged by GvHD, such as cells of the skin, liver and gastrointestinal tract. It may be that this diverse arsenal of mechanisms gives MSCs an advantage over more conventional single-target therapeutic agents, especially against a disease such as GvHD, which itself is underpinned by complex pathology involving a multitude of cell types and pathways. However, this also makes it extremely challenging to comprehensively elucidate the mechanisms of action of MSCs, either in general or with respect to the treatment of GvHD in particular. An improved understanding of MSC mechanisms of action would be beneficial for the clinical community, as well as providing a basis for the development of *in vitro* potency assays, to help identify and address problems with MSC variability.

## Conclusion

A substantial body of evidence suggests that MSCs have a beneficial effect in treating SR-aGvHD. Recent innovations may be capable of overcoming problems associated with inter-donor variability and functional changes associated with extensive culture expansion. Further adequately powered prospective studies are required to confirm efficacy and establish the place of MSC therapy in the treatment of this condition.

Experience to date with MSCs as a treatment for chronic GvHD is much more limited. The prevalence of clinical investigation of MSCs for acute GvHD versus chronic GvHD might suggest that the clinical community has identified more promise in the former, but further investigation in chronic GvHD appears to be warranted.

## Author Contributions

KK and JR drafted and reviewed the manuscript. All authors contributed to the article and approved the submitted version.

## Funding

Cynata Therapeutics funded the production of the illustration in **Figure 1** and the manuscript publication fees. This work was supported by a National Health and Medical Research Council Investigator Grant #1177305 to JR for ‘driving clinical cell and gene therapy in Australia’. Additional support was provided by Cure the Future, Therapeutic Innovation Australia and an anonymous foundation to JR.

## Conflict of Interest

KK is an employee and shareholder of Cynata Therapeutics. JR has acted as a consultant for and received travel grants from Cynata Therapeutics.

The authors declare that this study received funding from Cynata Therapeutics. The funder was not involved in the study design, collection, analysis, interpretation of data, the writing of this article or the decision to submit it for publication, outside of the contributions of author KK.

## Publisher’s Note

All claims expressed in this article are solely those of the authors and do not necessarily represent those of their affiliated organizations, or those of the publisher, the editors and the reviewers. Any product that may be evaluated in this article, or claim that may be made by its manufacturer, is not guaranteed or endorsed by the publisher.
